# MDGRAPE-4: a special-purpose computer system for molecular dynamics simulations

**DOI:** 10.1098/rsta.2013.0387

**Published:** 2014-08-06

**Authors:** Itta Ohmura, Gentaro Morimoto, Yousuke Ohno, Aki Hasegawa, Makoto Taiji

**Affiliations:** Laboratory for Computational Molecular Design, RIKEN QBiC (Quantitative Biology Center), 6F, 1-6-5, Minatojima-minamimachi, Chuo-ku, Kobe, Hyogo 650-0047, Japan

**Keywords:** molecular dynamics simulation, high performance computing, multiscale simulation, scalable parallel system, hardware accelerator

## Abstract

We are developing the MDGRAPE-4, a special-purpose computer system for molecular dynamics (MD) simulations. MDGRAPE-4 is designed to achieve strong scalability for protein MD simulations through the integration of general-purpose cores, dedicated pipelines, memory banks and network interfaces (NIFs) to create a system on chip (SoC). Each SoC has 64 dedicated pipelines that are used for non-bonded force calculations and run at 0.8 GHz. Additionally, it has 65 Tensilica Xtensa LX cores with single-precision floating-point units that are used for other calculations and run at 0.6 GHz. At peak performance levels, each SoC can evaluate 51.2 G interactions per second. It also has 1.8 MB of embedded shared memory banks and six network units with a peak bandwidth of 7.2 GB s^−1^ for the three-dimensional torus network. The system consists of 512 (8×8×8) SoCs in total, which are mounted on 64 node modules with eight SoCs. The optical transmitters/receivers are used for internode communication. The expected maximum power consumption is 50 kW. While MDGRAPE-4 software has still been improved, we plan to run MD simulations on MDGRAPE-4 in 2014. The MDGRAPE-4 system will enable long-time molecular dynamics simulations of small systems. It is also useful for multiscale molecular simulations where the particle simulation parts often become bottlenecks.

## 1. Introduction

Proteins, nucleic acids and the other biological molecules act as complex molecular nanomachines that are designed by evolutionary processes. Studies on the biological functions of these molecules have key importance in drug design, disease analyses and bioengineering. Recent advances in structural biology have revealed the structural basis of biological molecules and have enabled various computational analyses on them. Among these methods, molecular dynamics (MD) simulation is one of the most important methods for studying the thermodynamic and dynamic properties of molecules, because they are flexible, complex and fluctuating [1]. In MD simulations, the trajectories of all atoms or metaparticles are tracked using Newton's equation of motion, and physical quantities such as free energies are calculated from these trajectories. The forces between particles are approximated using ‘force field’ parameters [2,3]. Many new algorithms have been developed to decrease the computational costs and to accelerate relaxations; however, the computational costs of MD simulations are still high. A lot of effort has been made to accelerate them on high-performance computers, including GPU-based accelerators [4–7].

**Figure 1.**
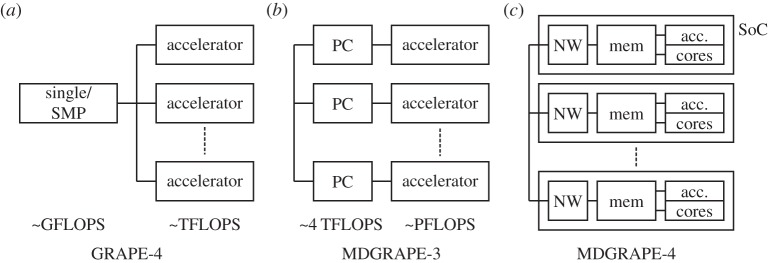
Transitions in GRAPE architecture. In TFLOPS GRAPE-4 (*a*), the single node could serve as the host, whereas the cluster is necessary for PFLOPS MDGRAPE-3 (*b*). For MDGRAPE-4 (*c*), integration to SoC was used to overcome a bottleneck that was due to the host system.

To accelerate MD simulations, several groups have tried, since the 1980s, to develop hardware accelerators [8–10]. We have continuously developed a series of special-purpose accelerators for particle simulations known as GRAvity PipE (GRAPE) [11–15]. The development of the GRAPE systems started with accelerators for astrophysical *N*-body simulations, and they have been extended for MD simulations such as MDGRAPE [16]. In this paper, we report on the architecture of the fourth-generation special-purpose computer for MD simulations, MDGRAPE-4. The MDGRAPE-4 is a successor to MDGRAPE-3 [17,18], but the architecture has been changed dramatically. Figure 1 illustrates the architectural transitions of GRAPE/MDGRAPE systems. The GRAPE-4 for astrophysics, which was completed in 1995, was the first tera floating-point operations per second (TFLOPS) machine [14]. It used a single processor or symmetric multiprocessor (SMP) workstation as the host machine. At that time, the performance of the host processor and the performance of the accelerator processor were almost equal at approximately 0.6 GFLOPS. The GRAPE-4 has the standard GRAPE-style architecture—the accelerator performs the dominant part of the calculation, and the rest of the calculations are performed on the host workstation. The MDGRAPE-3, which was completed in 2006, was the first PFLOPS machine [18]. The speed of the MDGRAPE-3 accelerator chip was 200 GFLOPS, which was 10 times faster than the 20 GFLOPS speed of the host processor. Because accelerators are easily parallelized, the potential performance gain from accelerators is larger than that from the general-purpose (GP) processor. In addition, owing to the differences between the algorithms for MD and *N*-body, MD requires a more powerful host computer. Thus, we needed to use a PC cluster as the host machine for MD. As a result, the communication latency and bandwidth became bottlenecks in the performance scaling as the accelerator performance increased. A typical system size for protein simulations is approximately 10^5^ atoms, which requires a computational cost of approximately 5 G operations per timestep. This means that the elapsed time to perform a single simulation step is only 5 μs when using a machine with sustained PFLOPS-level performance. Obviously, it is quite difficult to achieve this type of performance using accelerators that are attached to the I/O buses of a PC cluster.

Shaw's [19] research designed a special-purpose computer named Anton that solved this problem using a system on chip (SoC) that integrated specialized pipelines, GP cores, memory banks and networks. By decreasing latencies between the various elements, they achieved high scalability—15.4 μs per step for the 23 k atom system [20]. The MDGRAPE-4 takes a similar approach and uses a SoC to integrate the functions of a host and an accelerator into a single chip. Because the complexity of the design is greatly increased when compared with the MDGRAPE-3 large-scale integration (LSI) chip, we tried to keep the system as simple as possible. The development effort was also enabled by recent developments in semiconductor intellectual property cores for processor cores and high-speed serial interfaces.

We plan to finish the MDGRAPE-4 in 2014. The SoC and the test board have already been delivered. This paper describes the architecture of the MDGRAPE-4 system. In §2, the algorithm of the MD simulation is overviewed. In §3, we present the hardware architecture of MDGRAPE-4. The software and estimated performance are described in §4, and future directions are discussed in §5.

## 2. Basic algorithm of molecular dynamics simulations

### (a) Basic interaction in molecular dynamics

In a classical MD simulation, each atom is assumed to be a point mass obeying Newton's equation of motion
2.1
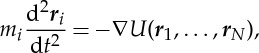

where *U* is the potential energy, *N* is the number of atoms, *m*_*i*_ is the mass and ***r***_*i*_ is the coordinate of the *i*th particle. At each discrete timestep, the position of each atom is updated by numerical integration of the equation (2.1). The potential *U* is approximated using a force field. For example, in the popular Amber force field the potential field, the potential is expressed by
2.2
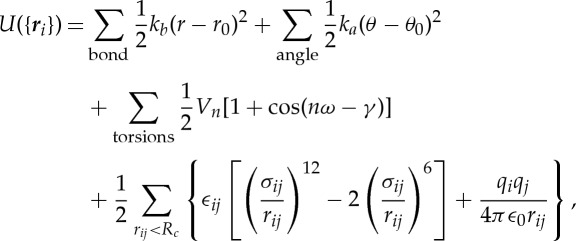

where *r*_*ij*_=|***r***_*i*_−***r***_*j*_|[21]. In equation (2.2), the first three terms express bonded forces, and the last summation describes non-bonded forces. Note that bonded forces such as bond stretching, angle and torsion refer only to several topologically connected atoms; therefore, their computational costs are relatively lower than that for non-bonded forces. When the number of atoms is *N*, the calculation costs of numerical integration for bonded forces are proportional to *N*, but the cost of direct calculation for non-bonded force is proportional to *N*^2^, which is more expensive. To reduce this calculation cost, we use well-established numerical algorithms, such as the particle mesh Ewald (PME) method [22], to calculate the long-range Coulomb force efficiently by decomposing the force into a short-range part and a long-range part.

### (b) Particle mesh Ewald

PME is an efficient method for calculating lattice sums with periodic boundary conditions. In the Ewald method, short-range interactions are calculated directly as pairwise interactions and long-range interactions are calculated using the Ewald summation, a kind of lattice-sum, on the periodic boundary condition in reciprocal space [23]. In the Ewald method, the Coulomb potential *U*_C_ is decomposed as
2.3
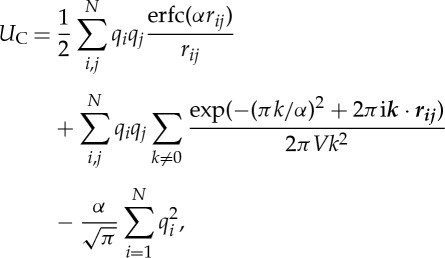

where *α* is the constant proportional to the inverse of the Gaussian width. The second term is the Ewald summation. To enhance the convergence of the lattice sum, the charges of atoms are diffused by a Gaussian (or the other kernels) for the long-range interaction. After subtraction of the long-range Coulomb potential that is convoluted by the Gaussian kernel, the Coulomb potential is modified by a factor of the complementary error function erfc(*αr*) (the first term of equation (2.3)). Because this deformed potential decays rapidly, we can truncate it at a short cut-off length. At optimal *α*, the number of wavenumber decreases smaller than *N*, and the cost of the discrete Fourier transformation decreases smaller than *O*(*N*^2^), but the cost is still expensive for large systems. In the PME method, charges of atoms are assigned to mesh points, and the charge distributions on the mesh are converted to a reciprocal space using fast Fourier transform (FFT). Using PME, the calculation cost is reduced to the order of 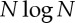. Although FFT limits the scalability of the PME method, it scales very well up to mid-sized parallel computers with a few thousand cores [4–6]. Because it is often the best solution for typical biomolecular systems with the modest numbers of atoms, the PME and its variants are the most widely used methods for the biomolecular MD simulations. Smooth charge assignment functions generate highly accurate results, but high costs are associated with them, because charges must be assigned to numerous mesh points. Therefore, many types of charge assignment functions have been proposed, such as Lagrange interpolate in the original PME, and B-spline in the smoothed PME [24]. Among them, Gaussian split Ewald (GSE) [25], which is also used in Anton, has a good property for an acceleration by specialized pipelines, because its Gaussian spread function has a spherical symmetry. We plan to use the *k*-GSE method in the MDGRAPE-4.

## 3. MDGRAPE-4 hardware

### (a) System overview

**Figure 2.**
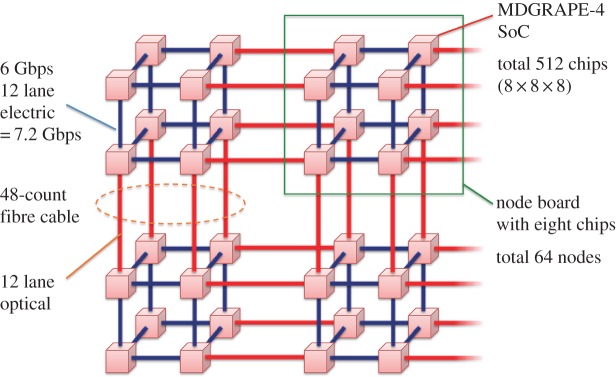
MDGRAPE-4 system. Red cubes represent SoCs. Blue and red lines between them are 12-lane, 6 Gbps, electrical and optical interconnects, respectively. The system consists of 512 chips in three-dimensional 8×8×8 torus network. A node consists of eight chips in a 2×2×2 cube topology. Gbps denotes gigabit per second.

**Figure 3.**
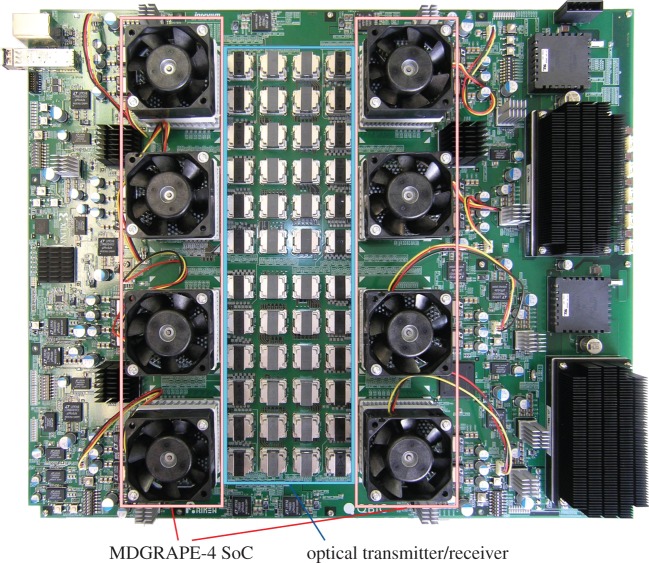
Photograph of an MDGRAPE-4 node board. It has eight MDGRAPE-4 SoCs and 24 optical transmitter/receiver pairs. The board size is 500 mm (width)×420 mm (height).

Here, we describe the MDGRAPE-4 system hardware. Figure 2 shows a diagram of the MDGRAPE-4 system. It consists of 512 SoCs connected by a three-dimensional, 8×8×8 torus network. The SoC was fabricated by the Hitachi HDL4S 40 nm CMOS technology. Because the SoC integrates processors, memory banks and networks into a single chip, it is the only major component, and no extra memory has been attached. Figure 3 shows a photograph of an MDGRAPE-4 node board with eight SoCs. The node forms a small 2×2×2 cube as shown in figure 2. In each direction, adjacent SoCs are connected by 12 lanes of full-duplex, 6 Gbps serializer/deserializer (SerDes). The SoCs on the same node are connected electronically, and the optical transmitter/receiver modules (Avago Technologies MiniPOD) are used for internode communication. For the internode connections, 48-count optical fibre cables (Fujikura Ltd) are used. The host PC needs to be able to communicate with the nodes for bootstrapping, controlling, monitoring and data I/O. Each node has five field-programmable gate arrays (FPGA, Xilinx Virtex-6 and Spartan-6) for these purposes. An optical, 6.5 Gbps, small form-factor pluggable (SFP) transmitter/receiver module is used for the host interface. It is connected to the host PC via an FPGA board (Tokyo Electron Device, TB-6V-SX475T-PCIEXP) on peripheral component interconnect express (PCI Express). Almost all the other parts on the board are power modules. The node board is mounted on an air-cooled 2U-height 19′′ standard EIA subrack. The total system consists of 64 nodes mounted on four pedestals. Another 19′′ pedestal is used for the host cluster. In the following, we describe the details of the SoC and the network.

### (b) System-on-chip architecture

**Figure 4.**
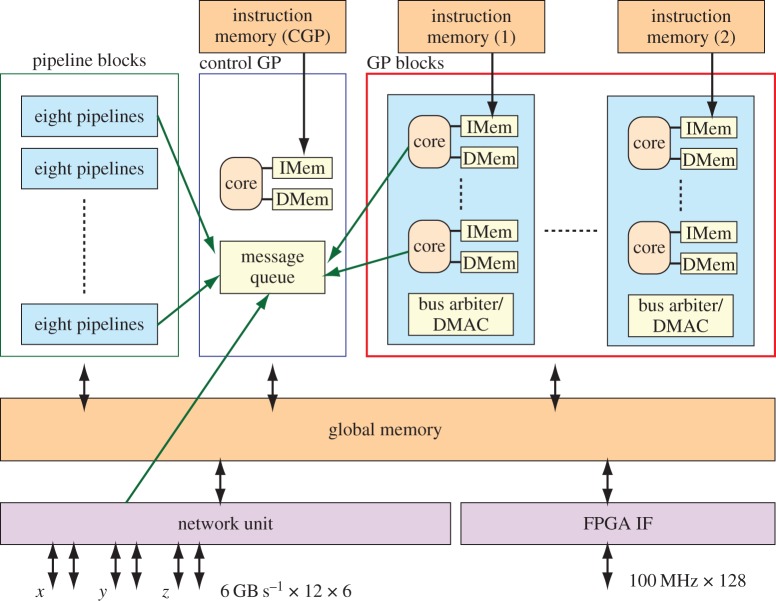
Block diagram of MDGRAPE-4 SoC. It has pipeline blocks, GP core blocks, control GP core, global memory, network units, FPGA interface and instruction memory banks. There are eight pipeline blocks with eight pipelines and eight GP blocks with eight GP cores.

Figure 4 shows a block diagram of the MDGRAPE-4 SoC. The MDGRAPE-4 chip has force calculation pipeline processors (PP), GP cores, a GP control core (control GP), network interface units (NIF), an FPGA interface and memory units. The force calculation pipeline unit accelerates the calculations for non-bonded particle-to-particle interactions. The GP cores are parallel GP cores. The number of processors is 64 for both the pipeline units and the GP cores, and each of them is grouped into eight blocks with eight units. The global memory (GM) is a 1.8 MB shared memory unit that is used to store data and instructions. The instruction memory of 512 KB can be used to feed instructions to the local instruction memory for each of the GP cores via direct memory access (DMA). Each SoC chip has two instruction memory banks, each of which is shared by four GP blocks (32 cores). They are protected with one-bit error correction/two-bit error detection for every 64 bits. The control GP core controls the flow of calculations. It communicates with the GP cores, pipelines and NIFs through queues prepared by Tensilica. The FPGA interface is a PCI-like bus controller with a 128-bit width. Its signal level is +1.2 V CMOS and its data rate is 100 MHz. The clock speeds are 0.8 GHz for the pipelines and 0.6 GHz for all other units. The voltages are +0.9 V for the SoC core, +1.2 V for I/O and +1.8 V for SerDes. The power consumption of the SoC is expected to be less than 65 W in the worst case.

**Figure 5.**
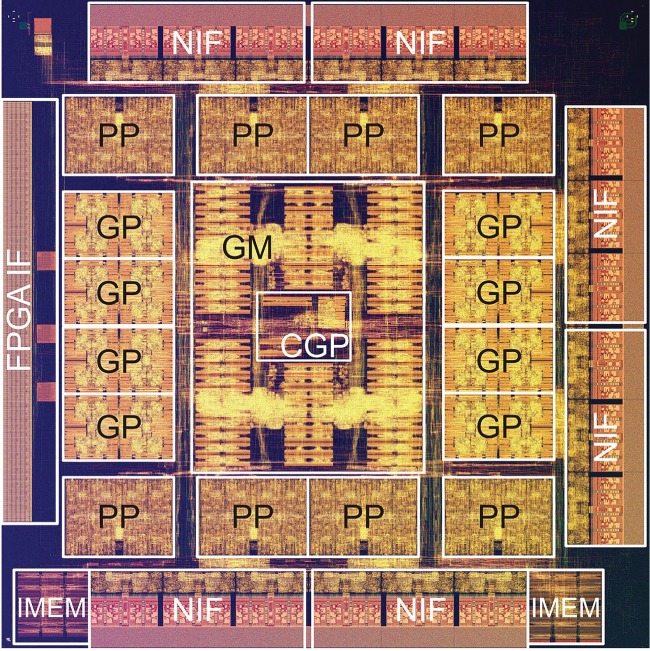
Physical layout of MDGRAPE-4 SoC. PP, pipeline block; GP, GP core block; GM, global memory; NIF, network interface; FPGA IF, FPGA interface; CGP, control GP core; IMEM, instruction memory. The image is provided by a courtesy of Hitachi Ltd.

Figure 5 shows the physical layout of the MDGRAPE-4 SoC. Its size is 15.6×15.6 mm^2^. Because most of the communications inside the chip are paired with the GM or the control GP core, they are placed in the centre of the die. The GP and pipeline blocks are located in the surrounding areas. The NIFs with the SerDes and the FPGA interface is placed at the edges. The pipeline dominated about 80% of the die area in the MDGRAPE-3 LSI. In contrast, about 20% of the die is used for the pipeline in the MDGRAPE-4.

### (c) Force calculation pipelines

The MDGRAPE-4 SoC has force calculation pipelines, which are dedicated hardware for two-body non-bonded interaction calculations. They calculate Coulomb and van der Waals forces and potentials expressed as
3.1
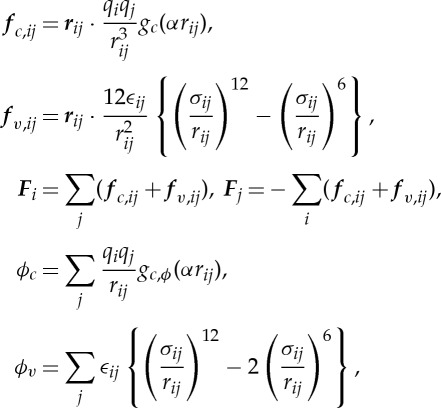

where ***r***_*ij*_=***r***_*i*_−***r***_*j*_, the suffixes *i* and *j* denote the atom number, the suffixes *c* and *v* denote Coulomb and van der Waals terms, respectively, and ***r***_*i*_ is the position of the *i*th particle. There are two parameters for Coulomb interaction, the charge *q*_*i*_ and cut-off coefficient *α*. The functions *g*_*c*_(*t*) and *g*_*c*,*ϕ*_ (*t*) are the cut-off functions for the Coulomb interaction that satisfy ***f***_*c*,*ij*_=−∇*ϕ*_*c*_. The two cut-off functions are prepared for the Gaussian kernel and the finite-support kernel.

The parameters for the van der Waals interactions, *ϵ*_*ij*_ for the potential depths and *σ*_*ij*_ for the radii, are calculated from the atom types. For *i* and *j* atoms, the coefficients *ϵ*_*i*_,*σ*_*i*_ and *ϵ*_*j*_,*σ*_*j*_ are obtained by table lookups. The maximum number of atom types is 64. The values of *ϵ*_*ij*_ and *σ*_*ij*_ are calculated as the arithmetic or geometric means of *ϵ*_*i*_,*ϵ*_*j*_ and *σ*_*i*_,*σ*_*j*_, respectively, based on the combination rule for the force fields.

The calculations are performed in mixed precisions and formats. Mostly fixed-point or logarithmic formats are used. The input coordinates are expressed in a 32-bit fixed-point format. The two-body interactions are evaluated at nearly equivalent precision with single-precision floating point. The force summation is performed in a 32-bit fixed-point format.

Although the MDGRAPE-4 force calculation pipeline has a structure that is quite similar to that of the MDGRAPE-3 pipeline [17,26], the following major differences exist. (i) MDGRAPE-4 calculates Coulomb and van der Waals force and potentials simultaneously, whereas the MDGRAPE-3 pipeline can evaluate only one of the four terms at a time. (ii) MDGRAPE-4 evaluates fixed function forms using an ROM table, whereas MDGRAPE-3 can evaluate flexible functions using RAM table. (iii) MDGRAPE-4 uses action–reaction symmetry (Newton's third law) to reduce the computational cost in half, whereas MDGRAPE-3 accumulates ***F***_*i*_, but not ***F***_*j*_, to minimize communication with the host computer. These design decisions were brought about by the increased speed and area cost of memory banks due to the process migration. The use of action–reaction symmetry is enabled by the integration of the GP cores, and the main memory banks that allows us to ignore the slow bandwidth to the host machine.

The pipeline has double-buffered registers that allow for the storage of sources and results for up to 16 particles, for both *i* and *j*. The eight pipelines form a pipeline block. The pipeline DMA controller transfers pipeline commands and particle data to the pipeline block. When the same particle data can be shared by several pipelines in the same block, the data are broadcasted to them. Similarly, when the forces on the same particle group are retrieved from several pipelines in the same block, the forces are summed before retrieval to the main memory. This parallelization method is referred to as broadcast parallelization [15]. Because it decreases the bandwidth to the main memory while maintaining parallelism, it is one of the important advantages of specialized architecture for particle simulations.

The pipelines operate at a speed of 0.8 GHz and they are asynchronous with the rest of the system. The MDGRAPE-4 SoC has 64 pipelines in eight blocks. Thus, it can handle a peak calculation load of 51.2 G interactions per second. Approximately 50 operations are required to perform the same calculation using general-purpose computers. Thus, the total peak performance of pipelines is approximately equivalent to 2.5 TFLOPS. It is no longer useful to compare the nominal FLOPS count with the FLOPS count for general-purpose computers; it is just to get a feel for its performance.

The MDGRAPE-4 pipeline has several extra functions in addition to the abovementioned basic functions. It can perform Gaussian charge assignment and back interpolation for Ewald mesh methods. Additionally, it supports soft-core van der Waals potentials and particle-group-based interaction cut-offs, which are useful for free-energy calculations.

### (d) General-purpose cores

In the MDGRAPE-4 SoC, we use the GP cores to process all tasks other than non-bonded force calculations, such as bonded-force calculations, time integrations, and FFT. The GP core used is a 0.6 GHz Xtensa^TM^ LX4-customized core that was developed by Tensilica Inc. [27]. The same core is used for the control GP core. Thus, there are 65 cores on each chip. Tensilica's core has many customizable options and extensions, and a hardware design is automatically generated based on a specification defined by the user. The GP core has extensions for a single-precision floating-point unit, a queue unit and a general-purpose I/O (GPIO) unit. There are extensions that affect performance such as a zero-overhead loop and the flexible length Instruction extension (FLIX), which is a kind of very long instruction word (VLIW) architectures. However, they are not implemented because of their impact on the core size and the clock speed. While the instruction set is based on a reduced instruction set computing (RISC) architecture, multiple instruction lengths are supported. This feature enlarges the size of the GP core about 20%; however, we implemented the function because it can save the capacity of the instruction memory banks and improve the efficiency of the local instruction cache and memory.

The GP core has both local memory banks and cache memory banks for flexibility of data placement and for a better utilization of the data/instruction localities that are essential for many-core architectures. It has 8 KB of data memory and 8 KB of instruction memory in addition to 4 KB of data cache and 4 KB of instruction cache. The latency to the GM can be hidden by the caches or by prefetching to the local memory banks using DMA. The size of the data cache allows for the storage of data for approximately 100 atoms. On the other hand, it is difficult to optimize the size of instruction cache. Because the efficiency of the set-associative cache depends heavily on the access patterns of the applications, we tried to estimate an optimal cache size by running MD simulations (GROMACS v. 4.5.4 [21]) using the software emulation environment of Tensilica. We particularly focused on the instruction cache miss in long loops that is executed on the GP cores and estimated the sufficient size of 4 KB to vanish the instruction cache misses within long loops.

In addition to the caches, the core also has local memory banks to store reusable data. Each core has 8 KB of a data memory and an instruction memory of the same size. Instructions in the local instruction memory are sent using manual DMAs from the on-chip instruction memory. The DMA data bus has a 64-bit width at 0.6 GHz, and it can broadcast the same instructions to all eight cores in the same GP block. The instruction fetch is double-buffered, so that the instruction DMA will not stall instruction executions. The shared instruction memory is not mapped to the address space of the core, and access to it is not cached, because it was difficult to implement the alternate path to it on the processor core. The data memory can also be accessed by a DMA from the GM unit in addition to the normal direct access. The DMA unit is shared by the eight cores in the same GP block, and the DMA bus width is 64-bit at 0.6 GHz. All these local and cache memory banks have 32-bit parity for error detections.

### (e) Global memory

**Figure 6.**
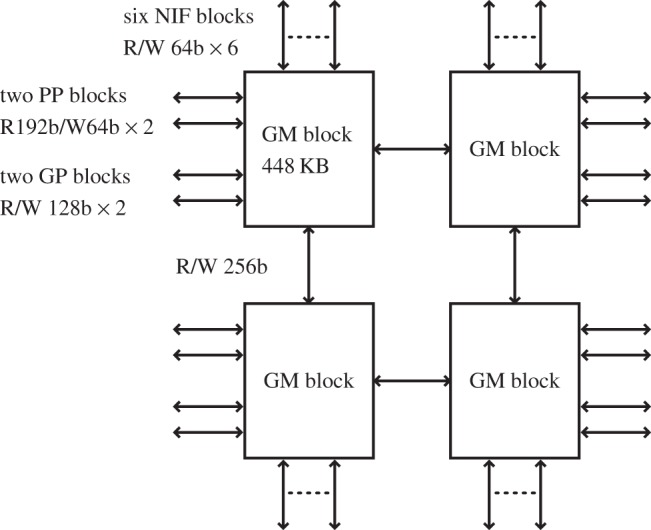
Global memory. It has four GM blocks, each of which has 448 KB of memory and linked with two GP blocks, two pipeline blocks and all six NIF blocks. They are also connected by a ring bus.

MDGRAPE-4 has a 1.8 MB shared memory known as GM. It is implemented physically by four of the same units owing to difficulties in physical implementation of a many-port memory unit. Each unit has a capacity of 448 KB, which consists of 28 pages of 16 kB memory with 32-bit parity. Figure 6 illustrates the GM and its interfaces.

The four GM units are connected by a ring bus with a 256-bit width, 19.2 GB s^−1^. Each unit has four read/write ports with a width of 64 bits for the two GP blocks and two ports with 192-bit read/96-bit write for the two pipeline blocks. It also has six ports with a width of 64 bits to communicate with six NIF units. The bandwidths of the GM units are 9.6 GB s^−1^, 14.4 GB s^−1^ (read)/7.2 GB s^−1^ (write) and 4.8 GB s^−1^ for the GP block, pipeline block and NIF unit, respectively. Consequently, the aggregate bandwidth to all the GP cores is 76.8 GB s^−1^, that to all the pipeline units is 115.2 GB s^−1^ (read)/57.6 GB s^−1^ (write), and that to all six NIF units is 28.8 GB s^−1^. The GM unit has a function for the accumulation of data. During DMA write from the pipelines, GP cores and network units, the written data can be summed up with data in the memory banks, rather than being written over existing data. The format of the accumulated data is limited to a 32-bit integer only. This function can decrease the cost of synchronization.

### (f) MDGRAPE-4 network

The MDGRAPE-4 system consists of a three-dimensional torus network which consists of six embedded network channels on the chip. The network channels are controlled by six NIF units for the (*X*,*Y*,*Z*)×(+,−) directions. Each unit has three SerDes blocks, each of which has four lanes with full-duplex 6 Gbps speed. The SerDes encodes the packets using 8b/10b coding. Therefore, the bandwidth between two interconnected chips is 7.2 GB s^−1^.

To send packets, the control GP unit manages the NIF units through the command sequence stored on the GM. The NIF creates packets by adding a header and a trailer based on the data from the GM via DMA. When the NIF receives packets from another chip, it interprets the packets and writes the content to the GM based on the packet header. Finally, it sends a signal about the received packet to the message queue of the control GP.

**Figure 7.**
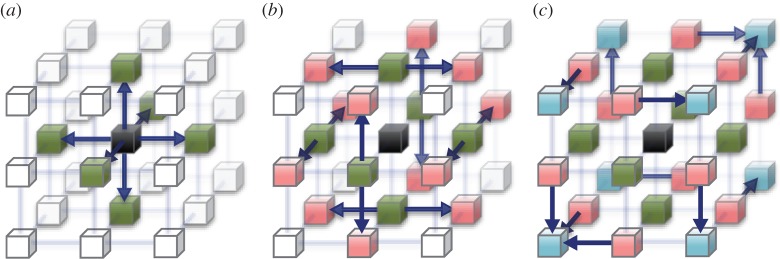
Routing pattern of the type II packets. To distribute the data on the central chip (black) to the surrounding 26 chips, three stages of forwarding on the torus network are required. The NIF forwards the type II packets to other directions according to the inward direction and the stage. The outward direction can be programmed by writing to the NIF register. To exploit the bandwidth of the network, evenly using six NIFs with less redundancy would be desirable. (*a*) At first, packets are transferred to the six nearest neighbours (green). (*b*) At the second stage, 12 chips (red) receive the packets. (*c*) At the third stage, eight chips in the diagonal position (blue) receive the packets. Two chips receive the packets with the same contents redundantly in this routing pattern.

The typical behaviour of the NIF (type I) is as described above. In addition, the NIF has routing mechanisms for sending packets to distant chips without mediation by the control GP unit. In MD simulations, space decomposition is usually used for massive parallelization, where each node has atoms in a rectangular parallelepiped slice. Thus, each chip has to distribute/gather data to/from the surrounding 26 chips. To reduce the latency of such communication, the type II packets are forwarded in the orthogonal directions after the first transfer. Figure 7 shows the routing pattern for the distributing of the data on a chip to surrounding chips.

Another routing mechanism gathers data in one direction. The type III packets are sent to the next chip in the same direction with a decrementing hop number, and they are acknowledged as being received when the hop number is zero. Using the packet routing and accumulation function of the GM, data from each chip in the communication line can be gathered and stored after reduction on a specified chip.

## 4. Software for MDGRAPE-4

Here, we describe the software developments and the preliminary estimated performance of the MDGRAPE-4 system. The control GP and the other GP cores are programmed using C and the assembly language. The operations of PPs and NIFs are instructed by command sequences placed in the GM. Currently, we are porting the ‘mdrun’ program within GROMACS [21], a versatile software package to perform molecular dynamics, for the MDGRAPE-4 system. The program supports several force fields and many algorithms such as the types of long-range electrostatic interaction, temperature or pressure couplings and bond constraints. The porting of the code is not straightforward, and many modifications are necessary. In particular, parallelization schemes should be redesigned for the MDGRAPE-4 system. In addition, each MDGRAPE-4 chip has only a small amount of memory for codes and no generic file access, the SoCs perform only the simplified main loop for MD simulations that support a force field, Ewald mesh method for long-range interaction, and leap-frog integrator. The other parts that have no serious effect on performance, the initialization and the file output for example, are executed by the host computers. The minimum plan of software support includes the all-atom MD with AMBER force field, periodic boundary condition, length/position constraints and temperature/pressure controls. In addition, we like to include the other functions like free-energy calculations. The porting of GROMACS is ongoing in collaboration with Prof. Lindahl's group, Stockholm University. The details of GROMACS on MDGRAPE-4 will be given elsewhere.

### (a) Coordination of calculations in the system on chip

This section describes the organization of the tasks by the many-core SoC. The control GP facilitates calculation processes; the control GP routinely checks messages on the queue from other on-chip units such as the GP cores, pipeline units and NIF units. It conducts these units to perform their tasks with proper timing. To invoke calculations in the pipeline units, the control GP sends a start address and a length of a command sequence to a pipeline control unit. The pipeline control unit fetches and analyses a command in sequence, and it sends the command header, the atom positions, charges, atom types and other necessary information read from the GM to the specified pipeline block. After the calculations are completed, the pipeline block returns results directly to the GM, and it also sends a message representing the endpoint of the pipeline calculations to the control GP based on the request in the command.

**Figure 8.**
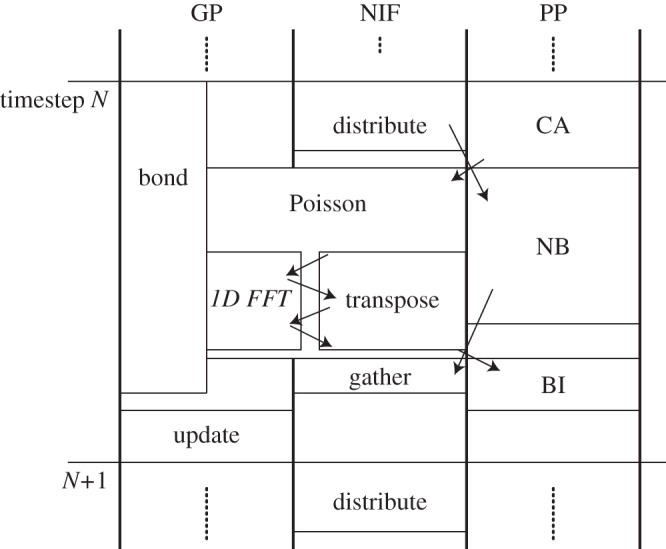
Calculation diagram of each unit at one timestep. The left column represents the task of GP cores (GP). The centre column is for NIF, and the right is the task of pipeline processors (PP). ‘Bond’ and ‘NB’ is the calculation of bonded force/non-bonded force. ‘CA’, ‘BI’, ‘distribute’, ‘gather’ and ‘update’ represents charge assignment, back interpolation, particle data distributing, force gathering, and update of the particle coordinates, respectively.

The control GP also checks for messages from the network. The control GP will receive a message from the NIFs when a packet with a message flag arrives. Based on this message, the control GP invokes the next calculations or transactions in the GP cores, the pipelines or the NIFs. Similarly, the completion of calculations in the GP cores will also be reported by a message to the queue of the control GP and vice versa. The control GP can send a message to the GP cores signalling the completion of the network transfers, pipeline calculations, and so on. Figure 8 illustrates the calculation flow for one timestep in an MD simulation.

At the first stage of each timestep, each chip distributes particle data to the surrounding 26 chips. In the Ewald mesh methods, calculations of long-range interactions require the following three stages: (i) charge assignments (CA), (ii) Poisson solver on a mesh, and (iii) back interpolation (BI). At the charge assignment stage, the charges of each atom are assigned to the mesh points by the pipelines. At the second stage, the GP cores of the whole system communicate each other to solve Poisson equations by FFT. At the back interpolation stage, the pipelines calculate a force on each atom from the potentials of the neighbour mesh points. Non-bonded short-range forces (NB) and bonded forces that can be calculated at an arbitrary time after the particle distribution stage by the pipelines and the GP cores. The non-bonded forces on the atoms in the other chips are sent back. Using the forces accumulated in the GM banks, the GP cores update the particle coordinates and velocities in the final stage of each timestep.

### (b) Performance estimates

We are implementing and testing the simple MD code using GP cores, PPs and NIFs on a prototype system with 2×2×2 torus topology. Here, we mention performance estimates of the final MDGRAPE-4 system. Although such codes are still fragmented and preliminary, in the following, we try to estimate the performance from the current results. In the estimate, we suppose a system with 50 k atoms, 100 atoms per each node. Using a current commodity cluster, it takes more than several milliseconds to finish one timestep computation of such systems.

On the performance of the GP cores, the current efficiency of the arithmetic operations is about 15% for most of calculation codes. The GP cores mainly calculate the bonded forces and the integration. There are three types of bonded forces, namely bond stretching, angle bending and torsion. The theoretical numbers of floating point operations (FLOP) were counted for the calculations of three bond types (table 1). The average number of FLOP for an atom, 992.8, was obtained as a sum of the average number of each bond type multiplied with the corresponding number of FLOP. In the case of domain decomposition, the bond type distribution variates according to the ratio of water and protein atoms in each domain. In the above average, the distribution in protein-rich domains is used, because protein-rich domains have a higher number of bonded interaction and heavier computational loads than water-rich domains. In the case of 50 k atom simulation, each GP core will treat two atoms. Because the performance of the GP core is 0.6 GFLOPS, it takes about 8.3 μs to process the bond interaction of two atoms, if we assume efficiency of 20%. The calculation time of the bonded interactions can be overlapped and hidden by that of non-bonded interactions. The integration costs little and is expected to finish in a few microseconds. The incorporation of the constraints and the temperature/pressure controls will affect the performance, which we have not evaluated yet.

**Table 1.** Estimated number of floating point operations for bonded force calculation. The fractional numbers appear for torsion, because each number of FLOP count is also averaged on torsion periods.

	arithmetic type							
FLOP count	add 1	mul 1	rsqrt 10	cos 10	acos 10	total FLOP	avg. num. per atom	total FLOP per atom
bond type								
bond stretching	12	9	1			31	1	31
angle bending	34	42	4	1	1	136	1.8	244.8
torsion	72.47	91.37	2			183.84	3.9	717.0
total								992.8

The tasks of the PPs are charge assignment to the mesh points (CA), calculation of the non-bonded interactions (NB) and back interpolation on the particles (BI). By a naive implementation, 100×1728 particle–mesh interactions and 100×100×63 non-bonded interactions are calculated by 64 pipelines. The current measurement of the pipeline efficiency is about 30%, and the PP works at 0.8 GHz. According to the measurements, the estimated time to finish is 10 μs, 30 μs and 10 μs for CA, NB and BI, respectively. Note that there is room to improve the performance by the optimization of command sequences.

On the NIFs, we measured the time to transfer some type I and type III packets and estimated the latency and the effective bandwidth. The results are summarized as *T*(ns)=600+450×*N*_hop_+200×*D*/128, with the number of hops (*N*_hop_) and the packet size (*D*) in four-byte words. Although the bandwidth is lower than expected (under investigation), we can estimate the time to distribute the particle data and to gather the forces based on the measurements. The size of the particle array is six words per atom and the size of the force array is three words per atom. Those should be transferred to the neighbours those are three hops distant. Then, it is estimated to be about 5 μs to distribute the particles and 4 μs to gather forces.

Three-dimensional FFT incorporates the coordination of NIF and GP tasks and is complicated to perform. Here, we estimate the time to solve 32×32×32 points FFT. By using the two-dimensional decomposition (64 nodes are involved), one-dimensional FFT of 32 points (three times) and axis transposition (twice) are interleaved. The FLOP count of the 32-point small FFT is 400 and it will finish in 3.3 μ*s*. To transpose the axis, at least 64 words of data must be transferred to the four hops distant node and it will take 2.5 μs. When solving the Poisson equation, forward and inverse three-dimensional-FFT are executed. In addition, the charges on mesh points are gathered at the beginning and the potential data are distributed at the end. Thus, we expect the time to solve the Poisson equation to be 35 μs.

These network performances estimated above can be optimistic, because the packet conflicts in NIFs are not taken into account. The series of the evaluation of the long-range interaction (CA, Poisson, BI) will probably be the critical path of the calculation. However, the long-range interaction is not always evaluated if multiple time step integration is incorporated. In this case, the series of the non-bonded interaction (particle distribution, non-bonded force, force gathering) will be critical. In our current estimate, it is hard to achieve less than 50 μs per one timestep in a simple simulation. The development of the software has just started, and we are trying to improve the command sequence to shorten the tasks and the control sequence to overlap the execution of the tasks.

## 5. Discussion

Here, we discuss software developments and future perspective to improve performance. First, it must be mentioned that one of the major differences between the software on an MDGRAPE-4 and that on a general-purpose machine is that there is no operating system within the MDGRAPE-4 SoC. To simplify the architecture and the GP cores, there is no virtualization of the memory address. Therefore, a pointer address indicates a physical address of the memory in the MDGRAPE-4. Programmers have to indicate the explicit data placement in different memory layers of the SoC—the local data memory in each core, the shared GM banks with a direct connection and those with indirect connections. It should also be mentioned that the cache memories of the GP cores have no coherency. Again programmers have to explicitly control the status of the GM and the cache. There are three ways to do this: a DMA write, a write to the shadow address range without caching and a cache flushing. In the case of the first two ways, the cache line should be invalidated for a next read access. The programming language is C or C++, but many features of C++ are not useful because of the limitations in the memory size, the memory allocations and the cache controls.

There is room for a discussion on the optimal number of divisions of the GM. The unified memory architecture is better for the usability and accessibility of the GM from a software point of view. On the other hand, it requires large multiplexers and increases latencies. If we divide the GM into smaller units, then the hardware complexity and the latency to the nearest memory bank will both decrease; however, latencies to the far memory banks will increase and bandwidths to them will decrease, thus more attention should be paid to the data placements. Further software analysis will answer the optimal configuration of the memory layers.

Because the calculation time per step has already reached the order of 10 μs, further improvement is getting harder and harder, and more specializations will be required to decrease the time. Another direction is to improve a cost performance. In future, the performance for a typical protein MD simulation saturates even with several chips. Systems with a single chip or a single module on a silicon interposer with several chips will be future solutions at a low cost and a low power with high performance.

## 6. Summary

The MDGRAPE-4 is a special-purpose computer system for MD simulations of proteins. It is based on many-core SoC technology that integrates all the elements of a high-performance computer, such as GP cores, memory banks and network interfaces, into a single LSI chip. It also has dedicated pipelines for non-bonded force calculations. The clock speeds of the SoC are 0.8 GHz for the pipelines and 0.6 GHz for the other units, and the power consumption of the SoC is designed to be less than 65 W, in the worst case. The SoCs are connected to each other by an interconnect with a peak bandwidth of 7.2 GB s^−1^ that forms a three-dimensional torus network with 512 (8×8×8) chips. The expected maximum power consumption is 50 kW. We aim to use this integration to accelerate the simulations of small systems with approximately 100 k atoms.
